# Supplementary material for: Beam damage of single semiconductor nanowires during X-ray nanobeam diffraction experiments
Source: J Synchrotron Radiat. 2020 Aug 12;27(Pt 5):1200–8. doi: 10.1107/S1600577520009789 (PMC7467348; doi:10.1107/S1600577520009789)
Supplement: Supplementary file 1 [file s-27-01200-sup1.pdf]

JOURNAL OF SYNCHROTRON RADIATION

Volume 27 (2020)

Supporting information for article:

Beam damage of single semiconductor nanowires during X-ray nano beam diffraction experiments

Ali AlHassan, Jonas Lähnemann, Arman Davtyan, Mahmoud Al-Humaidi, Jesús Herranz, Danial Bahrami, Taseer Anjum, Florian Bertram, Arka Bikash Dey, Lutz Geelhaar and Ullrich Pietsch

Part 1: He implementation

In order to replicate the nano X-ray diffraction (nXRD) experiment under He atmosphere, a cylinder made from kapton tape was used to shield the sample from the ambient conditions inside the experimental hutch. A picture of this configuration is shown in Fig. S1. A constant He flux enters through the blue tube and leaves through a hole in the side of the cylinder so that a constant pressure is maintained inside the cylinder.

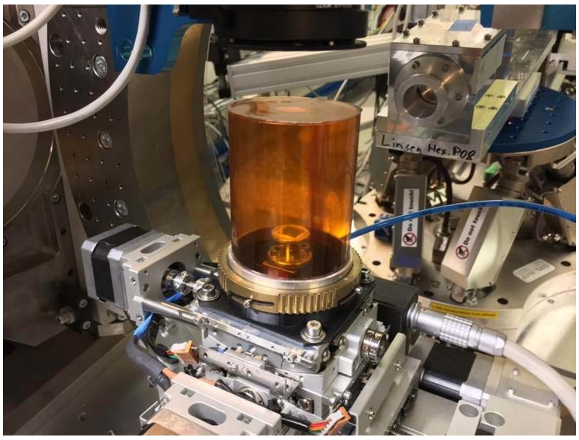

Figure S1: Kapton tape cylinder implemented to shield the sample from air atmosphere. The He gas enters through the blue tube. A hole was drilled at the side wall of the Kapton tape to release the He pressure.

Part 2: Reciprocal space maps for NW1

NW1 was exposed to X-rays for 1 hour in air. The reciprocal space maps (RSMs) recorded during this time are shown in Fig. S2 and show a variation of the Bragg peak along Q_Y^{111} only. Similar as during the first hour of exposure for NW2, the main peak splits into 2 sub-peaks P (peak) and T (tail). This behavior indicates that NW1 does not undergo any thermal expansion and moreover tilts only in one direction perpendicular to the [111] growth direction. Quantitatively, the NW is tilted by 0.4^{o} to 0.6^{o} from the initial orientation (Fig. S3).

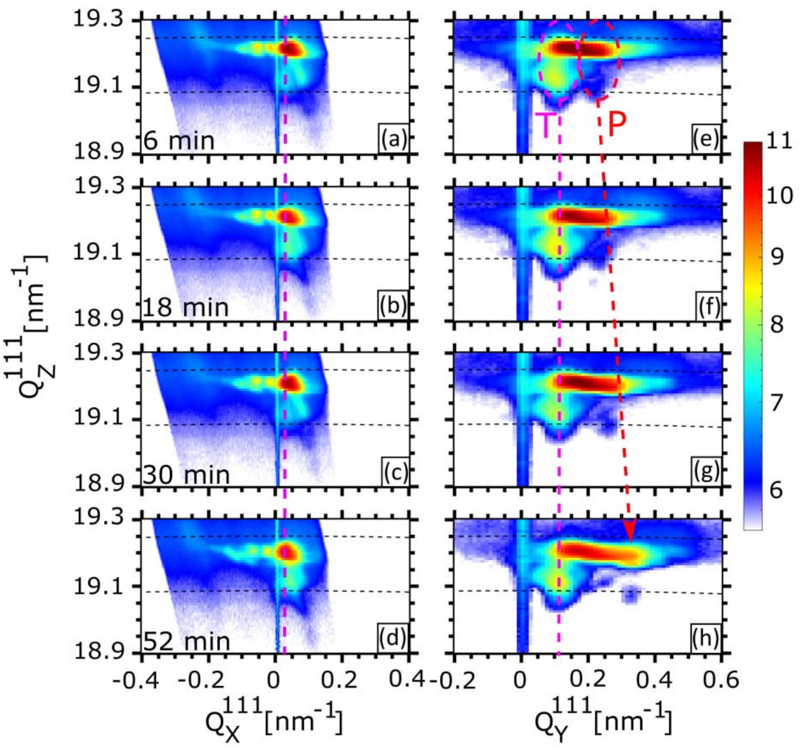

Figure S2: (a-d) RSMs in the (Q_Z^{111},Q_X^{111}) plane and (e-h) in the (Q_Z^{111},Q_Y^{111}) plane during exposure. The time at which the RSM acquisition was started is mentioned at the bottom left corner of each sub-plot. The pink dashed lines indicate the constant positions of T in Q_X^{111} (a-d) and Q_Y^{111} (e-h), whereas the red dashed arrow indicates the variation of P in Q_Y^{111} (e-h).

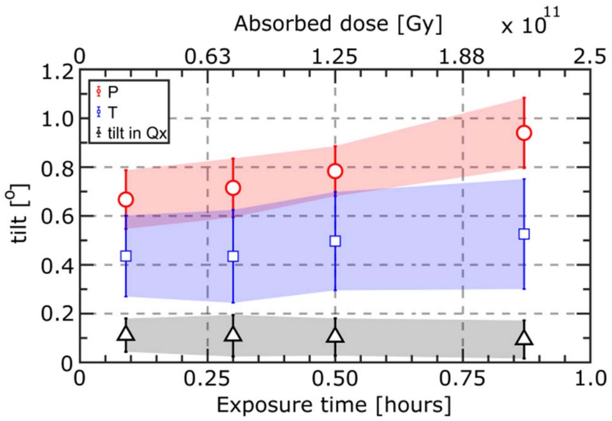

Figure S3: Tilt calculation of P and T (Q_Y^{111} direction), as well as in Q_X^{111} direction, calculated from the RSMs in Figure S2.

Part 3: Calculation of the absorbed dose

As shown in the main text, the structural changes within single nanowires (NWs) were monitored and displayed as a function of exposure time (hours) and absorbed dose rate (Gy). In order to

calculate the absorbed dose, first the cross-section of the experimentally used Gaussian nano-focused beam was reconstructed knowing that the total photon flux is 10^{10} s^{-1} and that the vertical and horizontal full width at half maxima of the beam are 600 nm and 1800 nm, respectively. Second, a single NW with nominal height of 2 μm and diameter of 150 nm was created and convoluted with the Gaussian beam as depicted in Fig. S4(a). The simulation considers a possible misalignment of the NW with respect to the beam during the experiment.

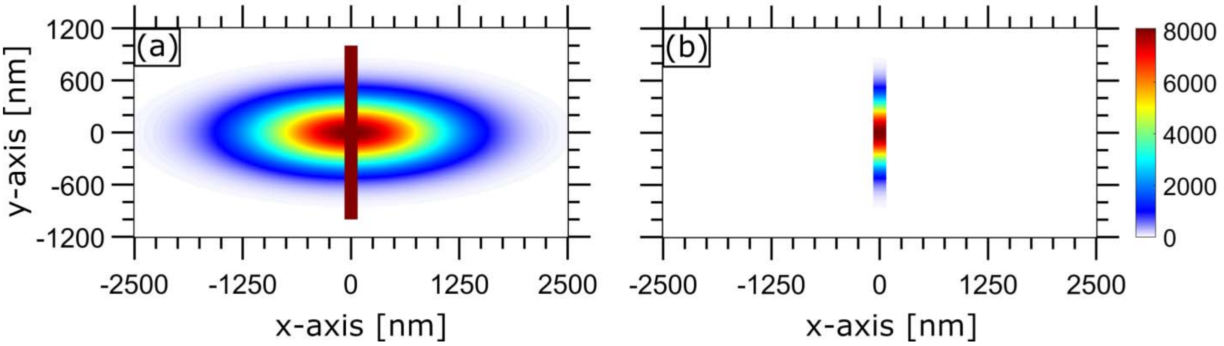

Figure S4: (a) The reconstructed Gaussian beam aligned to the center of a NW with nominal height of 2000 nm and diameter of 150 nm. (b) The number of photons illuminating the NW if the Gaussian beam hits the center of the NW.

Integrating the signal in Fig. S4(b), the photon flux that illuminates the NW (F^{ill}) is calculated to be 5.94×10^{8} s^{-1}. The dose is measured in Gy (J/kg), requiring the NW mass in kg and the deposited energy in J as input.

The total flux that hits the NW, expressed in units of J, depends on the number of photons with photon energy E_{ph} = 9 keV and is,

$$F^{\mathrm{tot}} = F^{\mathrm{ill}} \times E_{\mathrm{ph}} \times \mathrm{e} = F^{ill} \times 9 \times 10^{3} \times 1.6 \times 10^{-19}\ \mathrm{J} = 8.55 \times 10^{-7}\ \mathrm{J/s} \qquad (1)$$

Considering the nominal NW thickness of 150 nm and a height of 600 nm, given by the vertical full width at half maximum of the beam, the illuminated NW volume (V^{ill}) is calculated to be 1.17×10^{7} nm^{3}. The mass, M, of the NW is calculated using V^{ill} and the density of GaAs (ρ^{GaAs} = 5318 kg/m^{3}) as

$$M = V^{\mathrm{ill}} \times \rho^{\mathrm{GaAs}} = 6.22 \times 10^{-17}\ \mathrm{kg}. \qquad (2)$$

The dose rate per second (D_0) that hits the NW is defined as,

$$D_0 = F^{tot}/M = 1.37 \times 10^{10} \text{ Gy/s.} \qquad (3)$$

The dose rate absorbed by the NW requires the NW thickness d, which is chosen to be the nominal NW diameter of 150 nm, and the linear absorption coefficient μ, which is 3.37×10^{-5} nm^{-1}. Using these parameters, the absorbed dose rate per second D_A and the transmitted dose rate per second D_T can be calculated using equations 4 and 5,

$$D_A = D_0 \times (1 - e^{-\mu d}) = 6.94 \times 10^{7} \text{ Gy/s.} \qquad (4)$$

$$D_T = D_0 \times e^{-\mu d} = 1.37 \times 10^{10} \text{ Gy/s.} \qquad (5)$$

Part 4: Estimate of sample heating due to growth of an oxide layer

Using equation (1) from the main manuscript, one may estimate the difference in temperature between a heated NW (T_{NW}) and the surrounding (T_0) through a heat resistor layer of thickness d. Using $P = F_{tot}$; an oxide layer of $d = 100$ nm and an approximated heat transfer coefficient $\lambda = 1$ W/mK (Yoshikawa *et al.* 2013, Wingert *et al.* 2016) for the amorphous oxide shell, as well as the illuminated area equal to the total spot size (150 nm × 600 nm), we estimate

$$T_{NW} - T_0 = \frac{Pd}{A\lambda} = \frac{(9\times10^{-7}W)(10^{-7}m)}{1\frac{W}{mK}(9\times10^{-14})m^2} \approx 1K$$

This number is too small to explain a significant NW heating. However, considering that the major damage is found at the center of the Gaussian beam, A is reduced by a factor 100 to an area of about 900 nm² and the temperature change increases to 100 K. If the true photon flux is higher than estimated or the heat transfer coefficient is even lower, this number increases further. Thus, we can infer that NW melting can become possible for oxide thicknesses on the order of several ten nanometers.

Fig. S5(a) shows a sketch of the NW core-shell configuration and Fig. S5(b) the temperature increase for NW2. The temperature change presented in Fig. S5(b) has been calculated from the variation in the axial lattice variation (Δc) of WZ, ZB1, ZB2 and ZB3 in Fig. 6(d), where the

reference position corresponding to $\Delta T = 0$ is that of the main ZB and WZ Bragg peaks in Fig. 4(a). As demonstrated, the temperature can reach values above the congruent decomposition temperature of GaAs of approximately 680 ºC (Cheyn *et al.* 2015) and close to the melting point of GaAs of approximately 1240 ºC (Dhanaraj *et al.* 2010) with an error of about ± 230 ºC calculated by error propagation taking into account the uncertainties of the axial lattice parameter.

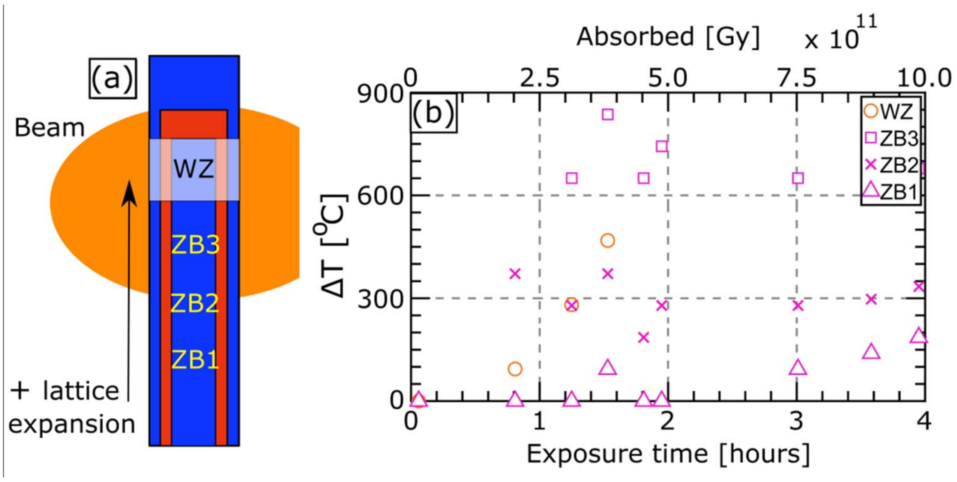

Figure S5: (a) Side view sketch showing the core-shell-shell configuration of a single NW. These NWs typically grow in the ZB crystal phase, whereas the WZ polytype is present in the upper part of the NW, just below the top section formed by axial elongation during shell growth. (b) Change in lattice temperature with exposure time (or absorbed radiation dose) extracted from the positions of the WZ and ZB Bragg reflections.

References:

Dhanaraj, G., Byrappa, K., Prasad, V. & Dudley, M. (2010). *Springer Handbook of Crystal Growth*. Springer-Verlag Berlin Heidelberg.
DOI: 10.1007/978-3-540-74761-1

Hey, Ch. & Jesson, D. E. (2015). *Appl. Phys. Lett.* 107, 161601.
DOI: 10.1063/1.4934218

Wingert, M. C., Zheng, J. , Kwon, S. & Chen, R. *Semicond. Sci. Technol.* **31**, 113003 (2016). DOI: 10.1088/0268-1242/31/11/113003

Yoshikawa, T., Yagi, T., Oka, N., Jia, J., Yamashita, Y., Hattori, K., Seino, Y., Taketoshi, N., Baba, T. & Shigesato, Y. (2013). *Appl. Phys. Express* **6**, 021101.
DOI: 10.7567/APEX.6.021101
